# A genome-wide association study of contralateral breast cancer in the Women’s Environmental Cancer and Radiation Epidemiology Study

**DOI:** 10.1186/s13058-024-01765-1

**Published:** 2024-01-23

**Authors:** Xiaohui Sun, Anne S. Reiner, Anh Phong Tran, Gordon P. Watt, Jung Hun Oh, Lene Mellemkjær, Charles F. Lynch, Julia A. Knight, Esther M. John, Kathleen E. Malone, Xiaolin Liang, Meghan Woods, Andriy Derkach, Patrick Concannon, Jonine L. Bernstein, Xiang Shu

**Affiliations:** 1 Department of Epidemiology and Biostatistics, Memorial Sloan Kettering Cancer Center, New York, NY 10017 USA; 2 Department of Epidemiology, Zhejiang Chinese Medical University, Zhejiang, China; 3 Department of Medical Physics, Memorial Sloan Kettering Cancer Center, New York, NY USA; 4 Diet, Cancer and Health, Danish Cancer Institute, Strandboulevarden 49, 2100 Copenhagen, Denmark; 5 Department of Epidemiology, University of Iowa College of Public Health, Iowa City, IA 52242 USA; 6 Prosserman Centre for Population Health Research, Lunenfeld-Tanenbaum Research Institute, Sinai Health, Toronto, ON Canada; 7 Dalla Lana School of Public Health, University of Toronto, Toronto, ON Canada; 8 Department of Epidemiology and Population Health, Stanford University School of Medicine, Stanford, CA USA; 9 Department of Medicine, Division of Oncology, Stanford University School of Medicine, Stanford, CA USA; 10 Epidemiology Program, Division of Public Health Sciences, Fred Hutchinson Cancer Research Center, Seattle, WA USA; 11 Department of Pathology, Immunology and Laboratory Medicine, Genetics Institute, University of Florida, Gainesville, FL USA

**Keywords:** Contralateral breast cancer, Genetic factors, Genome-wide association study, Polygenic risk score, Chemotherapy

## Abstract

**Background:**

Contralateral breast cancer (CBC) is the most common second primary cancer diagnosed in breast cancer survivors, yet the understanding of the genetic susceptibility of CBC, particularly with respect to common variants, remains incomplete. This study aimed to investigate the genetic basis of CBC to better understand this malignancy.

**Findings:**

We performed a genome-wide association analysis in the Women’s Environmental Cancer and Radiation Epidemiology (WECARE) Study of women with first breast cancer diagnosed at age < 55 years including 1161 with CBC who served as cases and 1668 with unilateral breast cancer (UBC) who served as controls. We observed two loci (rs59657211, 9q32, *SLC31A2*/*FAM225A* and rs3815096, 6p22.1, *TRIM31*) with suggestive genome-wide significant associations (*P* < 1 × 10^–6^). We also found an increased risk of CBC associated with a breast cancer-specific polygenic risk score (PRS) comprised of 239 known breast cancer susceptibility single nucleotide polymorphisms (SNPs) (rate ratio per 1-SD change: 1.25; 95% confidence interval 1.14–1.36, *P* < 0.0001). The protective effect of chemotherapy on CBC risk was statistically significant only among patients with an elevated PRS (*P*_*heterogeneity*_ = 0.04). The AUC that included the PRS and known breast cancer risk factors was significantly elevated.

**Conclusions:**

The present GWAS identified two previously unreported loci with suggestive genome-wide significance. We also confirm that an elevated risk of CBC is associated with a comprehensive breast cancer susceptibility PRS that is independent of known breast cancer risk factors. These findings advance our understanding of genetic risk factors involved in CBC etiology.

**Supplementary Information:**

The online version contains supplementary material available at 10.1186/s13058-024-01765-1.

## Introduction

Women with breast cancer have a twofold to sixfold increased risk of developing a new primary cancer in the contralateral breast (CBC) compared with the risk of developing a first primary breast cancer among the general population [1]. Genetic factors play a critical role in CBC development, including germline pathogenic variants in *BRCA1/2, TP53, CHEK2,* and *PALB2* [2–4]. Previous studies have investigated individual common variants in high- or moderate-penetrance breast cancer susceptibility genes [5] or drug metabolizing genes [6] and reported associations of breast cancer susceptibility variants identified from genome-wide association studies (GWAS) [7] with CBC risk. Studies have further demonstrated a positive cumulative effect of genetic variants, i.e., the polygenic risk score (PRS), on CBC risk, using a limited number of SNPs [8], in high-risk populations [9] or with limited adjustment for covariates [10]. However, a comprehensive GWAS assessing the associations between common variants and CBC risk has not been reported.

To advance the understanding of the genetic susceptibility of CBC for a large and growing population of breast cancer survivors, we carried out a GWAS in the Women’s Environmental Cancer and Radiation Epidemiology (WECARE) Study and evaluated the association between the updated breast cancer PRS [11] and CBC risk.

## Methods

### Study participants

The WECARE Study is a multi-center, population-based case–control study of CBC conducted in two phases: the WECARE I Study (2001–2004) and WECARE II Study (2009–2012) [12, 13]. Due to the word limit, we described the study design and participants in details in Additional file 1. The final analytic data set included 2829 participants (1161 cases and 1668 controls) for the main analysis and 2483 (1017 cases and 1466 controls) for the PRS analysis involving non-Hispanic White women only.

### CBC GWAS analysis

The genome-wide association analysis was performed in the combined data of the WECARE I and II Studies. Details about genotyping, quality control and imputation could be found in Additional file 1. Conditional logistic regression models with adjustment for the top five principal components (PCs) and age at first breast cancer diagnosis were performed to test additive effects of genetic variants. Genome-wide statistical significance was determined by the threshold of *P* < 5 × 10^–8^ with *P* < 1 × 10^–6^ considered as suggestive significance. The functional annotation was performed using Functional Mapping and Annotation (FUMA) [14]. We applied the Sum of Single Effects (SuSiE) method to identify credible sets in each identified locus [15]. Stratified analyses were further performed, and heterogeneity was assessed using the likelihood ratio test for nested models.

### PRS analysis

We constructed a weighted PRS, consisting of the 313 known breast cancer risk susceptibility SNPs [11]. Genotyping data were available at 239 of the 313 loci and proxies were determined for 18 of the remaining 74 loci. Detailed information could be found in Additional file 1. The association of the PRS with CBC risk was assessed using the continuous PRS, per standard deviation (SD) of the PRS, and PRS categorized by median, and quartile cut points based on UBC controls. Multivariable adjusted rate ratios (RR) and corresponding 95% confidence interval (CI) were estimated by fitting conditional logistic regression models adjusted for age at first breast cancer diagnosis, the top five genetic PCs, and known or suspected CBC risk factors. Area under the curve (AUC) of receiver operating characteristic (ROC) curves for various nested models were compared using the DeLong test [16].

All analyses were performed using R v4.1.3 or SAS v9.4 (The SAS Institute, Cary, NC).

## Results

**Fig. 1:**
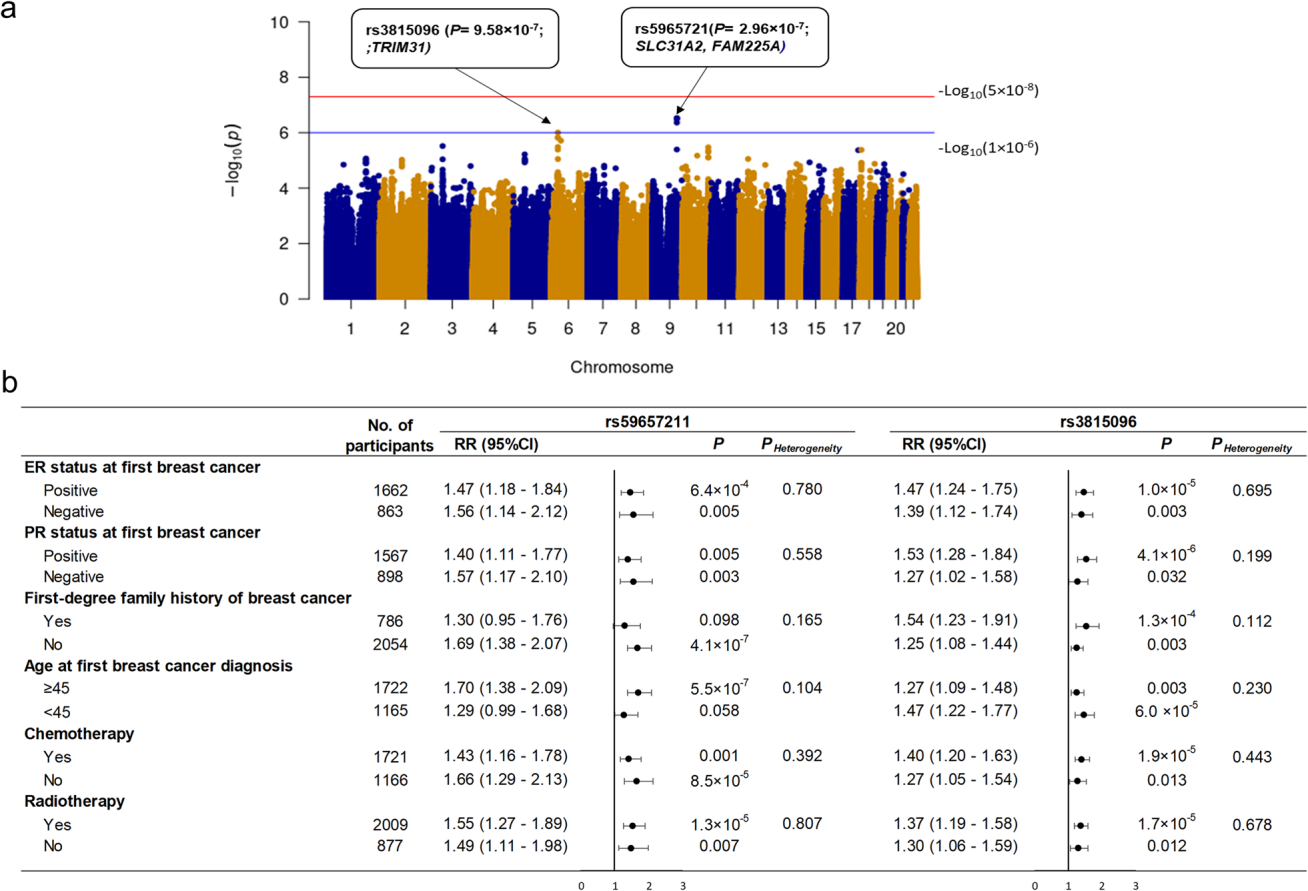
**a** Manhattan plot of GWAS for contralateral breast cancer risk in the WECARE STUDY; **b** stratified analyses for the two loci with suggestive genome-wide significant associations (*P* < 1 × 10^–6^) in the WECARE Study. CI, confidence interval; ER, estrogen receptor; No., number; PR, progesterone receptor; RR, rate ratio

The quantile–quantile (Q–Q) plot is shown in Additional file 2: Fig. S1. The inflation factor of the genome-wide scan was 1.034, indicating that the population structure was not an issue for the current analysis. Two loci associated with an elevated but not statistically significant CBC risk, 9q32 (rs59657211, *P* = 2.96 × 10^–7^, *SLC31A2/FAM225A*) and 6p22.1 (rs3815096, *P* = 9.58 × 10^–7^, *TRIM31*), were identified (Fig. 1a and Additional file 2: Fig. S2). One credible set, consisting of rs10817445, rs12337704, rs59657211, and rs9632905, was identified for 9q32 using SuSiE. However, SuSiE failed to identify any credible set for 6p22.1. There was no heterogeneity in associations of CBC with rs59657211 and rs3815096 by age at first breast cancer diagnosis, first-degree family history of breast cancer, ER and PR status, and chemotherapy or radiotherapy for the first breast cancer (Fig. 1b).

Among non-Hispanic White women, the weighted PRS without proxies (239 SNPs) was associated with an increased CBC risk of 46% (RR = 1.46, 95% CI 1.25–1.71 per weighted risk allele; RR = 1.25, 95% CI 1.14–1.36 per SD estimate). PRS were evaluated both as below and above the median and by quartiles; the above the median PRS category and the highest PRS quartile were both statistically significantly associated with increased CBC risk (Table 1). ROC curves were generated and AUCs were estimated to compare the discrimination ability of two models in the combined WECARE data (Table 2): CBC risk factors alone and PRS plus risk factors. The AUC of PRS plus risk factors model was 62.4 (95% CI 60.2–64.7), which was significantly higher than the model of the risk factors alone (AUC: 60.73, 95% CI 58.5–63.0, *P* = 0.01). We repeated the analysis in the WECARE I Study where information regarding *BRCA1/2* mutations was known. The AUC of model with PRS, risk factors, and *BRCA1/2* mutations was 67.7 (95% CI 64.7–70.7), significantly higher than the model with risk factors alone (AUC: 63.0, 95% CI 60.0–66.1, *P* < 0.0001) and the model with PRS plus risk factors (AUC: 65.1, 95% CI 62.0–68.1, *P* = 0.01). The association of PRS with CBC risk was modified by chemotherapy (*P*_heterogeneity_ = 0.04) such that the association between the PRS and CBC risk was stronger among women who did not receive chemotherapy for their first primary breast cancer compared to women who had chemotherapy (data not shown in Tables). When focused on the effects of chemotherapy, our data showed a reduced CBC risk among patients with higher PRS (RR = 0.61, 95% CI 0.46–0.81), but no association among patients with lower PRS (RR = 0.90, 95% CI 0.67–1.22) (Table 3). There was no heterogeneity in the association of the PRS with CBC risk by age at first diagnosis, family history of breast cancer, radiation treatment, ER status of first breast cancer, PR status of first breast cancer, hormone replacement therapy at first breast cancer, age at menopause, or parity (Table 3). Similar findings were observed when using the PRS with proxies (257 SNPs) (Table 1).

**Table 1:** Associations of weighted polygenic risk score comprised of known breast cancer susceptibility SNPs with contralateral breast cancer risk

PRS	CBCs No. (%)	UBCs No. (%)	RR^a^	95% CI^a^	*P*^a^
*Without proxies, 239 SNPs*					
Continuous weighted PRS	1017 (100)	1466 (100)	1.46	1.25–1.71	< 0.0001
Weighted PRS per Standard Deviation	1017 (100)	1466 (100)	1.25	1.14–1.36	< 0.0001
*Median of weighted PRS*					
Below median	415 (40.8)	733 (50)	Ref	Ref	
Above median	602 (59.2)	733 (50)	1.42	1.19–1.69	0.0001
*Quartile of weighted PRS*					
Quartile 1	192 (18.9)	366 (25)	Ref	Ref	Ref
Quartile 2	223 (21.9)	367 (25)	1.03	0.79–1.33	0.85
Quartile 3	260 (25.6)	366 (25)	1.27	0.99–1.63	0.06
Quartile 4	342 (33.6)	367 (25)	1.61	1.25–2.07	0.0002
*With proxies, 257 SNPs*					
Continuous weighted PRS	1017 (100)	1466 (100)	1.39	1.19–1.61	< 0.0001
Weighted PRS per standard deviation	1017 (100)	1466 (100)	1.22	1.11–1.33	< 0.0001
*Median of weighted PRS*					
Below median	416 (40.9)	733 (50)	Ref	Ref	
Above median	601 (59.1)	733 (50)	1.41	1.19–1.68	0.0001
*Quartile of weighted PRS*					
Quartile 1	211 (20.7)	367 (25)	Ref	Ref	
Quartile 2	205 (20.2)	366 (25)	0.87	0.67–1.12	0.28
Quartile 3	256 (25.2)	367 (25)	1.14	0.89–1.46	0.32
Quartile 4	345 (33.9)	366 (25)	1.50	1.18–1.92	0.001

Non-Hispanic white women only, WECARE I and WECARE II Studies combined

CBC, contralateral breast cancer; CI, confidence interval; No., number; PRS, polygenic risk score; RR, rate ratio; UBC, unilateral breast cancer

^a^Adjusted for the top five principal components, age at first breast cancer diagnosis, age at menarche, age at menopause, number of full-term pregnancies, stage of first breast cancer, histology of first breast cancer, family history of breast cancer, chemotherapy, and hormone therapy

**Table 2:** Areas under the receiver operating characteristic curve for contralateral breast cancer risk models

Model	AUC (95% CI)	AUC improvement (95% CI)	*P*
*WECARE I + II*			
Risk factors^a^	60.7 (58.5–63.0)	Ref	Ref
PRS + Risk factors	62.4 (60.2–64.7)	1.7 (0.4–3.0)	0.01
*WECARE I*^b^			
Risk factors	63.0 (60.0–66.1)	Ref	Ref
Risk factors + *BRCA1/2* mutations	65.5 (62.5–68.6)	2.5 (0.1–4.2)	0.003
PRS + Risk factors	65.1 (62.0–68.1)	2.0 (0.3–3.8)	0.03
PRS + Risk factors + *BRCA1/2* mutations^c^	67.7 (64.7–70.7)	4.7 (2.4–7.0)	< 0.0001

AUC, area under the curve; CI, confidence interval; PRS, polygenic risk score

^a^Risk factors included age at first breast cancer diagnosis, age at menarche, age at menopause, number of full-term pregnancies, stage of first breast cancer, histology of first breast cancer, family history of breast cancer, chemotherapy, and hormone therapy

^b^Information about *BRCA1/2* deleterious mutations was only available in the WECARE I Study

^c^When comparing PRS + Risk factors + *BRCA1/2* mutations to PRS + Risk factors model, the AUC improvement and *P* are: 2.2 (95% CI 0.5–3.8); 0.01

**Table 3:** Associations between estrogen receptor, progesterone receptor, family history of breast cancer, age at first breast cancer diagnosis, chemotherapy, radiotherapy, hormone replacement therapy, age at menopause, and parity and risk of CBC, stratified by high/low weighted PRS (239 SNPs) in Non-Hispanic White women

	Below median PRS				Above median PRS				
	CBCs No. (%)	UBCs No. (%)	RR (95%CI)	*P*	CBCs No. (%)	UBCs No. (%)	RR (95%CI)	*P*	*P*_*Heterogeneity*_
*ER status at first breast cancer*									
Positive	204 (54.9)	430 (65.5)	Ref		359 (70.1)	458 (73.6)	Ref		
Negative	168 (45.1)	227 (34.5)	1.33 (0.95–1.86)	0.09	153 (29.9)	164 (26.4)	1.11 (0.79–1.55)	0.55	0.41
*PR status at first breast cancer*									
Positive	194 (54.2)	408 (63.3)	Ref		339 (68.6)	440 (71.2)	Ref		
Negative	164 (45.8)	237 (36.7)	1.24 (0.88–1.74)	0.22	153 (31.4)	178 (28.8)	1.21 (0.87–1.68)	0.26	0.92
*Family history of breast cancer*									
No	284 (67.3)	592 (79.8)	Ref		384 (64.5)	544 (75.1)	Ref		
Yes	138 (32.7)	150 (20.2)	1.67 (1.23–2.25)	0.0009	211 (35.5)	180 (24.9)	1.66 (1.27–2.16)	0.0002	1
*Age at first breast cancer diagnosis*									
< 45	177 (41.9)	295 (39.8)	Ref		219 (36.8)	284 (39.2)	Ref		
≥ 45	245 (58.1)	447 (60.2)	0.66 (0.28–1.55)	0.34	376 (63.2)	440 (60.8)	0.66 (0.28–1.53)	0.33	0.96
*Chemotherapy*									
No	167 (39.6)	300 (40.4)	Ref		284 (47.7)	292 (40.3)	Ref		
Yes	255 (60.4)	442 (59.6)	0.90 (0.67–1.22)	0.51	311 (52.3)	432 (59.7)	0.61 (0.46–0.81)	0.0005	0.04
*Radiotherapy*^a^									
No	172 (40.8)	182 (37.2)	Ref		239 (40.2)	153 (37.3)	Ref		
Yes	250 (59.2)	559 (62.8)	1.05 (0.80–1.38)	0.71	356 (59.8)	571 (62.7)	0.95 (0.74–1.22)	0.67	0.59
*HRT at first breast cancer diagnosis*									
No	320 (76.4)	571 (77.5)	Ref		461 (78.3)	579 (80.3)	Ref		
Yes	99 (23.6)	166 (22.5)	1.08	0.67	128 (21.7)	142 (19.7)	1.22	0.25	0.59
			(0.76–1.53)				(0.87–1.70)		
Age at menopause^b^									
Postmenopausal 45+	106 (25.3)	153 (20.8)	Ref		145 (24.5)	168 (23.4)	Ref		0.46
Postmenopausal < 45	82 (19.6)	144 (19.5)	0.86	0.49	103 (17.4)	121 (16.8)	1.10	0.63	
			(0.57–1.32)				(0.74–1.65)		
Premenopausal	231 (55.1)	440 (59.7)	0.78	0.20	343 (58.1)	430 (59.8)	1.03	0.86	
			(0.54–1.13)				(0.73–1.46)		
*Parity at first breast cancer diagnosis*									
Parous	329 (78.3)	597 (80.6)	Ref		449 (75.8)	555 (77.0)	Ref		
Nulliparous	91 (21.7)	144 (19.4)	1.10	0.60	143 (24.2)	166 (23.0)	0.99	0.95	0.65
			(0.78–1.54)				(0.74–1.33)		

Non-Hispanic White women only, WECARE I and WECARE II Studies combined

CBC, contralateral breast cancer; CI, confidence interval; ER, estrogen receptor; No., number; PR, progesterone receptor; PRS, polygenic risk score; RR, rate ratio; UBC, unilateral breast cancer; HRT, hormone replacement therapy

Adjusted for the top five principal components, log-weight offset term, age at first breast cancer diagnosis, age at menarche, age at menopause, number of full-term pregnancies, stage of first breast cancer, histology of first breast cancer, family history of breast cancer, chemotherapy, and hormone therapy

^a^Control proportions account for counter-matched sampling of the WECARE I Study and do not represent true distributions in this population

^b^1 year prior to breast cancer diagnosis to preclude treatment-induced menopause

## Discussion

The present study is the largest population-based GWAS analysis of CBC risk to date and identified two loci with suggestive genome-wide significance. rs59657211 at the *FAM225A locus* has been reported to be involved in the tumorigenesis and metastasis of several types of cancers, including nasopharyngeal, colorectal, and esophageal squamous cell cancer [17, 18]. rs3815096, an intronic variant of *TRIM31,* is located within the major histocompatibility complex (MHC) region. It has also been reported to be nominally associated with risk of first primary breast cancer (OR 1.02, 95% CI 1.00–1.03, *P* = 0.007) in a prior GWAS [19], consistent with our results. *TRIM31,* a member of the TRIM family and acting as an E3 ubiquitin ligase, may play a promoting or suppressing role in malignant processes of multiple cancers [20, 21]. In breast cancer, *TRIM31* was found to suppress the cancer progression through the stabilization and activation of p53 [22]. Further investigation into these loci is needed to determine the underpinning mechanisms involved in CBC development.

We further confirmed an elevated risk of CBC is associated with the established breast cancer susceptibility PRS after the adjustment for other risk factors**.** Our findings corroborate prior studies that found a PRS consisting of the 313 breast cancer susceptibility SNPs associated with CBC risk [9, 10]. Moreover, the AUC that included the PRS and known breast cancer risk factors with or without BRCA1/2 mutations was significantly higher than that of the risk factors alone, suggesting the PRS may add additional predictive values in identifying breast cancer patients with an elevated risk of CBC. Our study also reported novel findings: i.e., chemotherapy was found to be protective among patients with higher PRS but was not among those with lower PRS. This suggests that breast cancer survivors with an unfavorable genetic background may benefit more from chemotherapy; when chemotherapy was not a viable option or the patients declined to receive the treatment, a more intense surveillance strategy might serve better for these patients for an early detection of CBC and treatment.

Our study has several strengths. Most notably, we included the largest number of CBCs reported in a GWAS study with available detailed risk factors, treatment, and clinical information. One primary limitation pertains to the generalizability across racial and ethnic groups as the WECARE Study included predominantly women of European ancestry and we lacked the statistical power to examine subgroups of interest.

In summary, our findings further the understanding of the genetic risk involved in CBC etiology, conferred by common SNPs. In turn, these results will be useful for the development of prevention strategies for CBC as well as for the long-term management of patients with breast cancer.

### Supplementary Information

**Additional file 1**. Description of methods for the current study.

**Additional file 2**. Supplementary Figures.

## Data Availability

Access to the WECARE data could be requested by submission of an inquiry to Dr. Jonine L. Bernstein (BernsteJ@mskcc.org) and WECARE Study Collaborative Group.

## Abbreviations

AUC: Area under the curve
CBC: Contralateral breast cancer
CI: Confidence interval
ER: Estrogen receptor
FUMA: Functional mapping and annotation
GWAS: Genome-wide association studies
HWE: Hardy-Weinberg equilibrium
LD: Linkage disequilibrium
MAF: Minor allele frequency
MHC: Major histocompatibility complex
OR: Odds ratio
PMRA: Precision medicine diversity array
PRS: Polygenic risk score
PR: Progesterone receptor
QC: Quality control
Q-Q: Quantile-quantile
ROC: Receiver operating characteristic
RR: Rate ratios
SNPs: Single nucleotide polymorphisms
SuSiE: Sum of single effects
TOPMed: Trans-omics for precision medicine
UBC: Unilateral breast cancer
WECARE: Women’s Environmental Cancer and Radiation Epidemiology
