# Supplementary material for: A genome-wide association study of contralateral breast cancer in the Women’s Environmental Cancer and Radiation Epidemiology Study
Source: Breast Cancer Res. 2024 Jan 23;26:16. doi: 10.1186/s13058-024-01765-1 (PMC10807183; doi:10.1186/s13058-024-01765-1)
Supplement: Supplementary file 1 — Additional file 1. Description of methods for the current study. [file 13058_2024_1765_MOESM1_ESM.docx]

**Supplementary Methods**

***Study participants***

In the WECARE Study, all participants were diagnosed with a first primary unilateral invasive local/regional breast cancer at age <55 years between 1985 and 2008 and were identified through six cancer registries in the US, one in Canada, and one in Denmark [1, 2]. Cases were subsequently diagnosed with an in situ or invasive CBC ≥ 1 year after the first diagnosis. Controls were women with unilateral breast cancer (UBC) who were individually matched to cases on year of birth (±5 year), year of first diagnosis (±4 year), cancer registry region, self-reported race and ethnicity, and no subsequent diagnoses of cancer during the interval between the matched case's first and second breast cancer diagnoses. All WECARE Study participants were interviewed by telephone using a structured questionnaire asking about personal demographics, medical history, cancer family history, menstrual and reproductive history, and hormone use. Data on treatment (chemotherapy, hormonal therapy, and radiation therapy) and tumor characteristics (estrogen receptor [ER] and progesterone receptor [PR] status, histology, stage) were collected from cancer registry records or by abstracting medical records [1-3]. The data collected from Danish participants were excluded due to regulatory issues relating to the General Data Protection Regulation, which were not resolved as of the writing of this manuscript. Informed consent was obtained from all participants in the study.

***CBC GWAS analysis***

DNA was extracted from either blood or saliva samples using standard methods and genotyping was performed using Illumina HumanOmni1-Quad v1.0 or Axiom Precision Medicine Diversity Array (PMRA) [1, 3, 4]. Pre-imputation quality control (QC) filtering by SNP/sample call rate < 95% and Hardy-Weinberg Equilibrium (HWE) *P* < 10^−7^ was performed with the exclusion of multi-allelic, duplicated non-SNP, and monomorphic sites. The imputation was performed using the Trans-Omics for Precision Medicine (TOPMed) genotype imputation server [5]. SNPs with imputation R^2^ < 0.3 or minor allele frequency (MAF) < 0.05 were excluded before downstream analyses. Consequently, 5,930,828 SNPs and 5,953,429 SNPs were retained in the WECARE I and II Studies, respectively. The identification of descent was examined using PLINK v2.0 [6]. The principal components for genome-wide association analysis were calculated by EIGENSTRAT [7]. In the present study, genetic variants with the lowest *P* at linkage disequilibrium (LD) < 0.1 in a LD block were considered as lead variants.

***Construction of PRS***

We constructed a weighted PRS, consisting of the 313 known breast cancer risk susceptibility SNPs [8].The proxies were determined (R^2^ > 0.8) using LDproxy [9]. The formula *PRS_i_=∑β_i_∗G_ij_* was used to construct the PRS, where *G_ij_* refers to the copy of risk alleles [reported odds ratio (OR) > 1] (or for imputed loci, the imputed dosage of values between 0 and 2, inclusive) and was weighted by the per-allele log odds ratio (*β_i_*) for each locus using published estimates [8]. For consistency with prior studies that generated the ORs used for the weighting, the PRS in this study was calculated for non-Hispanic White women.

**References**

1. Bernstein JL, Langholz B, Haile RW, Bernstein L, Thomas DC, Stovall M, Malone KE, Lynch CF, Olsen JH, Anton-Culver H *et al*: **Study design: evaluating gene-environment interactions in the etiology of breast cancer - the WECARE study**. *Breast Cancer Res* 2004, **6**(3):R199-214.

2. Langballe R, Mellemkjaer L, Malone KE, Lynch CF, John EM, Knight JA, Bernstein L, Brooks J, Andersson M, Reiner AS *et al*: **Systemic therapy for breast cancer and risk of subsequent contralateral breast cancer in the WECARE Study**. *Breast Cancer Res* 2016, **18**(1):65.

3. Brooks JD, Teraoka SN, Reiner AS, Satagopan JM, Bernstein L, Thomas DC, Capanu M, Stovall M, Smith SA, Wei S *et al*: **Variants in activators and downstream targets of ATM, radiation exposure, and contralateral breast cancer risk in the WECARE study**. *Hum Mutat* 2012, **33**(1):158-164.

4. Robson ME, Reiner AS, Brooks JD, Concannon PJ, John EM, Mellemkjaer L, Bernstein L, Malone KE, Knight JA, Lynch CF *et al*: **Association of Common Genetic Variants With Contralateral Breast Cancer Risk in the WECARE Study**. *J Natl Cancer Inst* 2017, **109**(10).

5. Kowalski MH, Qian H, Hou Z, Rosen JD, Tapia AL, Shan Y, Jain D, Argos M, Arnett DK, Avery C *et al*: **Use of >100,000 NHLBI Trans-Omics for Precision Medicine (TOPMed) Consortium whole genome sequences improves imputation quality and detection of rare variant associations in admixed African and Hispanic/Latino populations**. *PLoS Genet* 2019, **15**(12):e1008500.

6. Purcell S, Neale B, Todd-Brown K, Thomas L, Ferreira MA, Bender D, Maller J, Sklar P, de Bakker PI, Daly MJ *et al*: **PLINK: a tool set for whole-genome association and population-based linkage analyses**. *Am J Hum Genet* 2007, **81**(3):559-575.

7. Price AL, Patterson NJ, Plenge RM, Weinblatt ME, Shadick NA, Reich D: **Principal components analysis corrects for stratification in genome-wide association studies**. *Nat Genet* 2006, **38**(8):904-909.

8. Mavaddat N, Michailidou K, Dennis J, Lush M, Fachal L, Lee A, Tyrer JP, Chen TH, Wang Q, Bolla MK *et al*: **Polygenic Risk Scores for Prediction of Breast Cancer and Breast Cancer Subtypes**. *Am J Hum Genet* 2019, **104**(1):21-34.

9. Machiela MJ, Chanock SJ: **LDlink: a web-based application for exploring population-specific haplotype structure and linking correlated alleles of possible functional variants**. *Bioinformatics* 2015, **31**(21):3555-3557.
