# Supplementary material for: A genome-wide association study of contralateral breast cancer in the Women’s Environmental Cancer and Radiation Epidemiology Study
Source: Breast Cancer Res. 2024 Jan 23;26:16. doi: 10.1186/s13058-024-01765-1 (PMC10807183; doi:10.1186/s13058-024-01765-1)

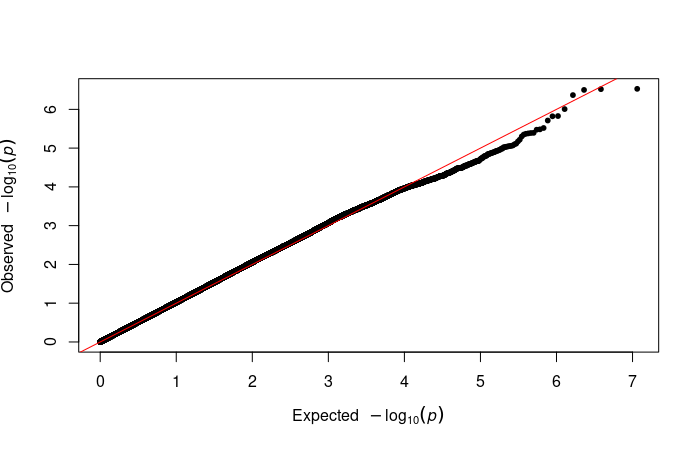
Supplementary Figure S1. The quantile-quantile plot of GWAS for contralateral breast cancer risk in the WECARE Study.

Supplementary Figure S2. Regional association plots of the rs3815096 and rs59657211 loci.


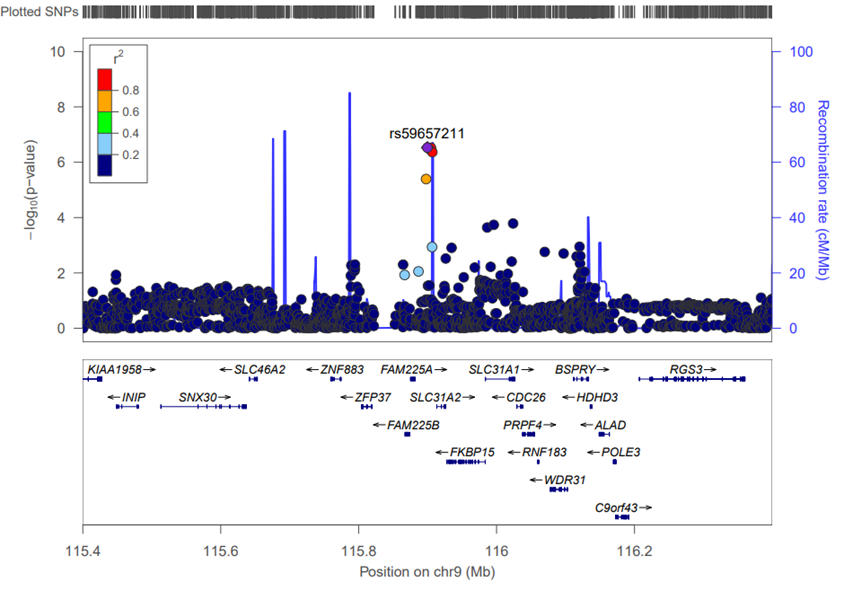

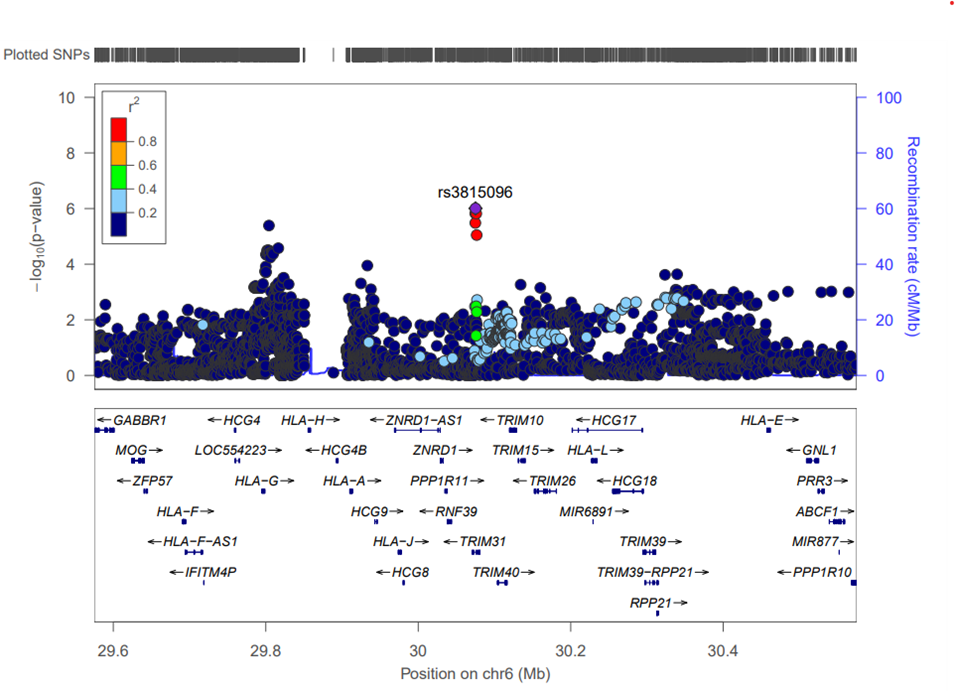

Supplement: Supplementary file 2 — Additional file 2. Supplementary Figures. [file 13058_2024_1765_MOESM2_ESM.docx]
